# Índice de Vulnerabilidade Social e Mortalidade por Doenças Isquêmicas do Coração e Doenças Cerebrovasculares no Brasil de 2000 a 2021

**DOI:** 10.36660/abc.20240428

**Published:** 2025-08-20

**Authors:** José Lucas Bichara, Luiz Antônio Bastos, Eric Delgado dos Santos Mafra Lino, Paolo Blanco Villela, Gláucia Maria Moraes de Oliveira

**Affiliations:** 1 Universidade Federal do Rio de Janeiro Rio de Janeiro RJ Brasil Universidade Federal do Rio de Janeiro, Rio de Janeiro, RJ – Brasil; 2 Instituto Militar de Engenharia Rio de Janeiro RJ Brasil Instituto Militar de Engenharia, Rio de Janeiro, RJ – Brasil

**Keywords:** Isquemia Miocárdica, Transtornos Cerebrovasculares, Doenças Cardiovasculares, Vulnerabilidade Social, Epidemiologia

## Abstract

**Fundamento:**

Estudos prévios observaram correlação entre as taxas de mortalidade por doenças isquêmicas do coração (DIC), doenças cerebrovasculares (DCBV) e o Índice de Vulnerabilidade Social (IVS). Porém, persistem dúvidas sobre a associação do IVS total e dimensões com a mortalidade estratificada por sexo, etnia e aglomerados populacionais.

**Objetivo:**

Analisar a evolução do IVS total e dimensões, e correlacionar com as taxas de mortalidade por DIC e DCBV no Brasil e Unidades da Federação (UF), de 2000 a 2021.

**Métodos:**

Estudo ecológico de séries temporais das taxas de mortalidade padronizada (método direto com a população brasileira de 2000) por DIC e DCBV por idade, sexo e UF entre 2000 e 2021 correlacionadas com o IVS e suas dimensões. Os dados das causas básicas de morte foram obtidos do Sistema de Informação de Mortalidade e os do IVS provieram do Atlas da Vulnerabilidade Social. Calculou-se correlação de Spearman (significativo se p<0,05) para cada estrato analisado.

**Resultados:**

O IVS, a dimensão capital humano (IVS-CH) e a dimensão renda e trabalho (IVS-RT) em 2010 apresentaram correlação forte com as variações das taxas de mortalidade por DCBV e DIC (IVS x DCBV: Rho(p)=0,85; IVS x DIC: Rho(p)=0,75; IVS-CH x DCVB: Rho(p)=0,84; IVS-CH x DIC: Rho(p)=0,84; IVS-RT x DCBV: Rho(p)=0,81; IVS-RT x DIC: Rho(p)=0,71. A dimensão infraestrutura urbana IVS-IU apresentou correlação fraca para DCBV e DIC, respectivamente (IVS-IU x DCBV: Rho(p)=0,33; IVS-IU x DIC: Rho(p)=0,25).

**Conclusão:**

O IVS-CH e IVS-RT apresentaram maior grau de correlação com as variações das taxas de mortalidade por DIC e DCBV.

## Graphical abstract

**Figura Central:**
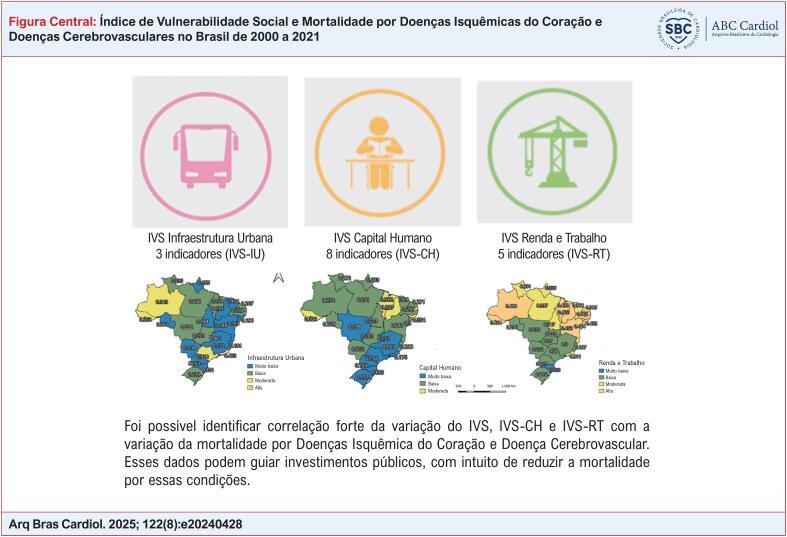
Índice de Vulnerabilidade Social e Mortalidade por Doenças Isquêmicas do Coração e Doenças Cerebrovasculares no Brasil de 2000 a 2021

IVS: Índice de vulnerabilidade social; IVS-IU: Dimensão infraestrutura urbana do Índice de Vulnerabilidade social; IVS-RT: Dimensão renda e trabalho do Índice de Vulnerabilidade social; IVS-CH: Dimensão capital humano do Índice de Vulnerabilidade social. Os dados expostos nos mapas são do ano de 2021.

## Introdução

Em 2021, se estimou que ocorreram mais de 9 milhões de mortes por doenças isquêmicas do coração (DIC) e quase 4 milhões de mortes por acidente vascular cerebral isquêmico no mundo.¹ e aproximadamente 75% dessas mortes foram registradas em países em desenvolvimento.^2 , 3^ No Brasil, as doenças cardiovasculares (DCVs) também são a principal causa de morte, representadas pelas DIC e a doenças cerebrovasculares (DCBV), em sua maioria.^2 - 6^ No final do século XX ocorreu redução da mortalidade por essas condições de modo mais acelerado em regiões com melhores indicadores socioeconômicos, como a Europa Ocidental e a América do Norte.^7^ No Brasil, as Unidades Federativas (UF) das regiões Sul, Sudeste e Centro-Oeste que têm os melhores indicadores socioeconômicos apresentaram maiores reduções das taxas de mortalidade por DIC e DCBV.^6 , 8 , 9^

Nos últimos anos, houve crescimento do número de estudos que associaram os indicadores socioeconômicos com as taxas de mortalidade pelas DCVs, entretanto, ainda são poucos os que empregaram os indicadores de vulnerabilidade nessa análise.^6 , 8 , 10 - 13^ Apesar disso, em comum, tais estudos identificaram maiores taxas de mortalidade em populações mais vulneráveis.^6 , 8 , 10 - 13^

O termo vulnerabilidade social possui diversas definições, uma das mais aceitas é "a ausência ou insuficiência de ativos que podem ser providos pelo estado".^14^ Não há uma padronização em âmbito global do índice de Vulnerabilidade Social (IVS). No Brasil, seu cálculo é realizado pelo Instituto de Pesquisa Econômica Aplicada (IPEA) que quantifica 16 indicadores alocados em 3 dimensões: Infraestrutura urbana, capital humano e renda, trabalho e forma de inserção (formal ou não). Por fim, se calcula a média aritmética dessas 3 dimensões para chegar ao valor do IVS total.^14^ A dimensão infraestrutura urbana tem por objetivo refletir o acesso ao saneamento básico e transporte urbano. Já a dimensão capital humano visa avaliar as condições de saúde e acesso à educação de uma população. Por fim, a dimensão renda, trabalho e forma de inserção almeja identificar a insuficiência de renda e de fluxo de renda.^14^ O IPEA disponibiliza ainda o cálculo do IVS e de suas dimensões para alguns segmentos específicos da população, como população feminina, masculina, negra, branca, rural e urbana. Esses dados podem auxiliar na identificação das características de maior fragilidade nas populações mais vulneráveis.

O objetivo deste artigo foi analisar a evolução do IVS total e suas dimensões, e sua associação com as taxas de mortalidade por DIC e DCBV, no Brasil e nas UF, no período de 2000 a 2021.

## Métodos

Estudo ecológico e descritivo das variações das taxas de mortalidade por DIC e DCBV em ambos os sexos e faixas etárias entre os anos de 2000 e 2021. Analisou-se também o IVS total e dimensões, no Brasil e nas UF, entre 2000 e 2021.

Os dados sobre as causas básicas de óbitos foram obtidos no site do Sistema de Informações sobre mortalidade (SIM) do Departamento de informática do Sistema único de Saúde (DATASUS) do Ministério da Saúde (MS).^15^ Foram selecionadas as informações sobre mortalidade referentes ao Brasil e em suas UF. Utilizaram-se como variáveis a faixa etária, o sexo e óbitos por residência. Para a pesquisa, a população foi estratificada em faixas etárias da seguinte forma: 0-19 anos, 20-29 anos e subsequentemente em faixas com 10 anos até o grupo de maiores de 80 anos.

Para seleção de óbitos cuja causa básica era DIC se utilizaram os códigos I20-I25 do Código Internacional de Doenças (CID-10). Para DCBV, os códigos são do I60 ao I69.^16^ Sequencialmente, se procedeu ao *download* de arquivos em formato .CSV que foram convertidos para XLS no programa Microsoft Excel, utilizado para análise de dados e construção de gráficos e tabelas.

As informações sobre a população residente no Brasil e em suas UF foram retiradas do *site* do DATASUS,^15^ que utiliza os dados oficiais censitários do Instituto Brasileiro de Geografia e Estatística (IBGE) de 1980, 1991, 2000 e 2010, projeções intercensitárias até 2012, e projeções populacionais de 2013 em diante.

As informações sobre o IVS foram obtidas do Atlas da Vulnerabilidade Social, disponibilizado pelo IPEA,^14^ onde foi obtido o valor do IVS e suas dimensões para a população total e para os estratos populacionais disponibilizados. O IVS foi considerado muito baixo quando era inferior a 0,2, baixo quando estava entre 0,2 e 0,3, médio quando estava entre 0,3 e 0,4, alto quando estava entre 0,4 e 0,5 e muito alto quando era maior que 0,5.^14^ Tais valores foram usados como referência para a coloração dos mapas e tabelas do material suplementar.

Foram realizados os cálculos das taxas de mortalidade bruta e da padronizada^17^ por faixa etária e sexo pelo método direto, tendo como população padrão a população brasileira do ano 2000 para a DIC e para a DCBV. Avaliou-se a tendência temporal das taxas de mortalidade padronizadas por faixa etária e sexo no período de 2000 a 2021, e a associação com o IVS e suas dimensões do ano de 2010. O ano de 2021 foi escolhido como o ano limite, pois é o ano mais recente com dados disponíveis para todas as variáveis analisadas.

Foram construídas tabelas usando os dados dos anos de 2000 e de 2021 para o IVS e suas dimensões. Para a construção dos gráficos foram utilizadas as variações percentuais das taxas de mortalidade padronizadas por DIC e DCBV e o IVS do ano de 2010. Utilizou-se uma defasagem temporal de aproximadamente 10 anos entre os indicadores e as taxas de mortalidade conforme estudos prévios sobre o tema.^8 , 18 , 19^ Para análise do grau de correlação foram realizados os cálculos para obtenção da correlação de Spearman (significativo se p<0,05) para cada estrato analisado. A correlação foi considerada fraca se ≤ 0,3, moderada se >0,3 e <0,7, e forte se ≥ 0,7.^20^

## Resultados

No período de 2000 a 2021, ocorreram 2.127.662 mortes por DCBV e 2.193.405 mortes por DIC, no Brasil, sendo 50,58% e 58,29% respectivamente, no sexo masculino. No mesmo período, o IVS do Brasil variou de 0,446 em 2000 a 0,249 em 2021.

**Figura 1:**
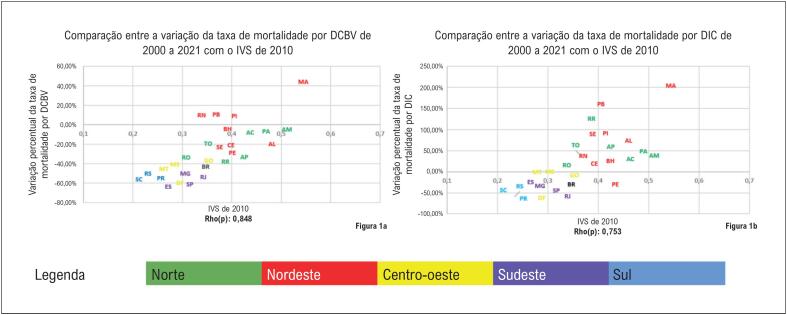
Comparação entre o IVS do ano de 2010 e a variação percentual da taxa de mortalidade por doenças cerebrovasculares e doenças isquêmicas do coração no Brasil e nas unidades federativas, em ambos os sexos, entre 2000 e 2021. (1A) - IVS de 2010 e variação percentual da mortalidade por DCBV, em ambos os sexos, no Brasil, 2000 a 2021, (1B) IVS de 2010 e variação percentual do da mortalidade por DIC, em ambos os sexos, no Brasil, 2000 a 2021.

A análise do IVS e de suas dimensões na população total e nos estratos populacionais disponíveis permitiu observar que houve melhora do indicador na quase totalidade das UF, entretanto durante todo período os melhores indicadores se concentram nas UF das regiões Sul, Sudeste e Centro-Oeste ( Figuras 1 e 2 e Tabelas 1,2,3 e 4 do material suplementar ). Além disso, se pôde observar que no ano de 2000 quase todas as UF com vulnerabilidade muito alta se localizavam nas regiões Norte e Nordeste do país ( Figura 1 e Tabelas 1,2,3 e 4 do material suplementar ). Outro fato a ser destacado é que as populações negra e rural apresentaram maior vulnerabilidade que os demais segmentos analisados ( Tabelas 1, 2, 3 e 4 do material suplementar ).

A dimensão infraestrutura urbana (IVS-IU) apresentou menor vulnerabilidade que o IVS total e o maior número de UF com vulnerabilidade baixa e muito baixa em 2021 ( Tabela 2 do material suplementar ). Maior número de UF foram classificadas como de vulnerabilidade muito alta na dimensão capital humano (IVS-CH), principalmente no ano de 2000, abrangendo todas as UF do Norte e Nordeste. Além disso, as populações negra e rural apresentaram vulnerabilidade maior do que os demais segmentos populacionais ( Tabela 3 do material suplementar ). Por fim, a dimensão renda e trabalho (IVS-RT) destacou a maior vulnerabilidade da população feminina em relação à masculina ( Tabela 4 do material suplementar ).

Na Figura 1 , se observa que a população das UF das regiões Norte e Nordeste teve maior vulnerabilidade e piora ou redução menos expressiva das taxas de mortalidade por DIC e DCBV. Destaca-se ainda que as UF da região Sul apresentaram menor vulnerabilidade com redução mais expressiva da mortalidade por DIC e DCBV. Para todos os cenários foi calculada a correlação de Spearman, tendo sido alcançado grau de correlação moderada a forte. Os coeficientes das correlações encontrados foram: IVS x DCBV: Rho(p)=0,85; IVS x DIC: Rho(p)=0,75.

**Figura 2:**
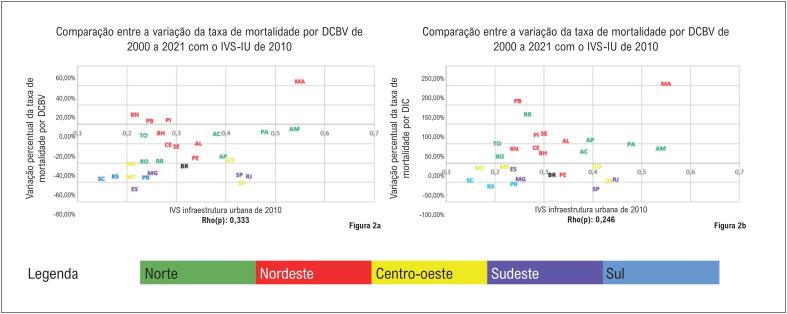
Comparação entre a dimensão de infraestrutura urbana do Índice de Vulnerabilidade Social de 2010 e a variação percentual da taxa de mortalidade por doenças cerebrovasculares e doenças isquêmicas do coração no Brasil e nas suas unidades federativas, em ambos os sexos entre 2000 e 2021. (2A) - IVS-IU de 2010 e variação percentual da mortalidade por DCBV, em ambos os sexos, no Brasil, 2000 a 2021, (2B) IVS-IU de 2010 e variação percentual da mortalidade por DIC, em ambos os sexos, no Brasil, 2000 a 2021.

Na Figura 2 se observa que as UF das regiões Norte e Nordeste apresentaram maior vulnerabilidade na dimensão infraestrutura urbana que as UF da região Sul. Além disso, as UF das regiões Norte e Nordeste apresentaram piora ou redução menos expressiva da mortalidade por DIC e DCBV. Entretanto, ao avaliar as regiões Centro-Oeste e Sudeste, se nota que Distrito Federal, Goiás, Rio de Janeiro e São Paulo apresentaram vulnerabilidade maior que a maioria das UF das regiões Norte e Nordeste. O cálculo da correlação de Spearman identificou correlação fraca. Os coeficientes das correlações encontrados foram: IVS-IU x DCBV: Rho(p)=0,33; IVS-IU x DIC: Rho(p)=0,25.

**Figura 3:**
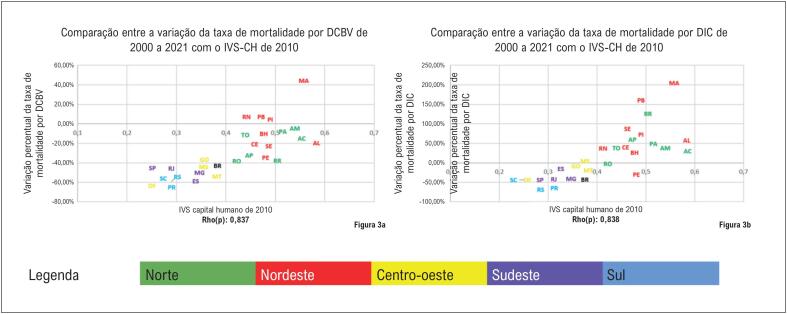
Comparação entre a dimensão capital humano do Índice de Vulnerabilidade Social de 2010 e a variação percentual da taxa de mortalidade por doenças cerebrovasculares e doenças isquêmicas do coração no Brasil e nas suas unidades federativas, em ambos os sexos, entre 2000 e 2021. (3A) - IVS-CH de 2010 e variação percentual da mortalidade por DCBV, em ambos os sexos, no Brasil, 2000 a 2021, (3B) IVS-CH de 2010 e variação percentual da mortalidade por DIC, em ambos os sexos, no Brasil, 2000 a 2021.

Na Figura 3 , se observou padrão semelhante ao identificado na Figura 1 , no qual as UF das regiões Norte e Nordeste apresentaram vulnerabilidade maior no quesito capital humano e piora ou redução menos expressiva das mortalidades por DIC e DCBV que as demais regiões. Interessante notar, que ao realizar o cálculo da correlação de Spearman, foi identificada correlação forte do IVS-CH comparada aos demais. Os coeficientes das correlações encontrados foram: IVS-CH x DCVB: Rho(p)=0,84; IVS-CH x DIC: Rho(p)=0,84.

**Figura 4:**
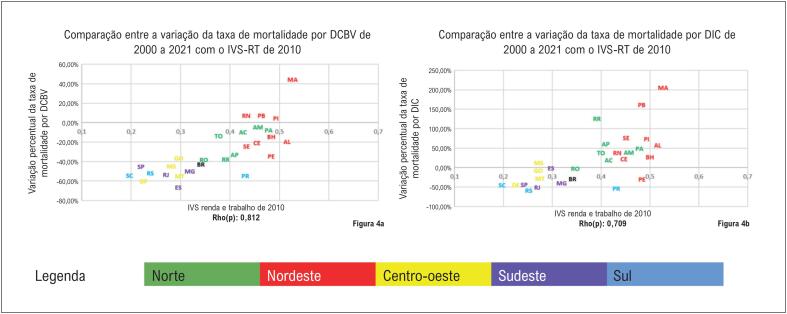
Comparação entre a dimensão de renda e trabalho do Índice de Vulnerabilidade Social de 2010 e a variação percentual da taxa de mortalidade por doenças cerebrovasculares e doenças isquêmicas do coração no Brasil e nas suas unidades federativas, em ambos os sexos, entre 2000 e 2021. (4A) - IVS-RT de 2010 e variação percentual da mortalidade por DCBV, em ambos os sexos, no Brasil, 2000 a 2021, (4B) IVS-RT de 2010 e variação percentual da mortalidade por DIC, em ambos os sexos, no Brasil, 2000 a 2021.

Na Figura 4 se nota novamente o padrão já descrito nas Figuras 1 e 3 . As UF das regiões Norte e Nordeste apresentam maior vulnerabilidade na dimensão renda e trabalho do IVS e apresentou ou aumento da mortalidade por DIC e DCBV, ou em caso de queda, esta foi menos expressiva quando comparada a outras UF das demais regiões. O cálculo da correlação de Spearman identificou correlação forte, entretanto de modo menos expressivo que os valores obtidos com o IVS-CH e o IVS. Os coeficientes das correlações encontrados foram: IVS-RT x DCBV: Rho(p)=0,81; IVS-RT x DIC: Rho(p)=0,71.

## Discussão

Na avaliação das dimensões do IVS se observou que apesar da melhora dos indicadores no período, a grande maioria das UF de Norte e Nordeste permaneceram com a maior vulnerabilidade do país em todas as dimensões. Além disso, foi evidente maior vulnerabilidade das populações negra e rural e maior vulnerabilidade na dimensão capital humano do IVS. Esses achados foram também descritos em outros estudos que observaram maior vulnerabilidade das populações negra^21 - 23^ e rural^24 - 26^ em diversas áreas, incluindo o acesso à saúde.

Observou-se que houve ou aumento da mortalidade por DIC e DCBV, ou em caso de queda, esta foi menos expressiva nas UF das regiões Norte e Nordeste quando comparada a outras UF, apesar da redução da vulnerabilidade observada nesse período. A análise das correlações da variação da mortalidade por DIC e DCBV com as dimensões do IVS isoladamente, permitiu identificar que o IVS-CH (>0,83- forte) alcançou um grau de correlação superior ao do IVS (>0,75 – forte). Além disso, o IVS-RT também apresentou correlação forte enquanto o IVS-IU teve correlação fraca (<0,35). Estudos prévios já haviam identificado a associação de indicadores socioeconômicos de desenvolvimento e vulnerabilidade com a mortalidade por essas condições,^6 , 8 , 9 , 18 , 27 - 31^ entretanto ainda não havia sido realizada uma análise para identificar quais fatores teriam maior influência.

Em relação à vulnerabilidade, estudos dos Estados Unidos da América sugeriram que as métricas de vulnerabilidade poderiam apresentar boa correlação com desfechos em saúde pública.^31^ Estudos internacionais identificaram que populações mais vulneráveis estão sujeitas a apresentar mais fatores de risco cardiovascular como hipertensão arterial sistêmica, dislipidemia, diabetes e tabagismo,^10 , 32^ maior dificuldade de acesso a serviços de saúde,^33^ menor acesso a serviços de reabilitação cardíaca,^33 , 34^ mais readmissões hospitalares em 30 dias por insuficiência cardíaca^35^ e aumento da mortalidade, inclusive mortalidade precoce por DCVs.^12 , 13^

Estudos prévios, que analisaram o IVS no Brasil, identificaram a melhora desse indicador em âmbito nacional, entretanto com maior vulnerabilidade das regiões Norte e Nordeste,^6 , 8^ compatível com nossos achados. Em relação a mortalidade por DCBV, também foi identificado uma redução da mortalidade nas UF das regiões Sul, Sudeste e Centro-Oeste,^6^ assim como para DIC.^8^ Este estudo sugere quais fatores socioeconômicos teriam maior influência sobre a mortalidade de DIC e DCBV.

A dimensão infraestrutura urbana, que avalia coleta de lixo, fornecimento de água e esgoto inadequados e tempo de deslocamento casa-trabalho, apresentou grau de correlação inferior às demais. Esses achados se devem principalmente ao fato das UF do Distrito Federal (DF), Goiás (GO), Rio de Janeiro (RJ) e São Paulo (SP) terem apresentado maior vulnerabilidade, diferenciando-se do padrão observado nas demais dimensões. Pode-se supor que no RJ e em SP isso se deva aos grandes movimentos migratórios a partir da década de 1950 que levaram a um rápido e desorganizado aumento da população urbana, culminando na formação das favelas.^36 , 37^ As UF de GO e DF também sofreram com processo semelhante, embora de menor dimensão, a partir da década de 60, motivados pela construção da Capital Federal e posteriormente pela expansão da fronteira agrícola.^38^

Outras hipóteses para o fato de as correlações não serem fortes para a IVS- IU, pode ser a necessidade de maior defasagem temporal entre a melhoria dessa dimensão da vulnerabilidade social para que ocorra a redução da mortalidade, ou ainda que essa dimensão tenha sido representada por outras do IVS capital humano, que mensura a mortalidade infantil e anos estudados, ou o IVS renda e trabalho com avaliação da renda domiciliar per capita, que isoladamente já mostram correlação em estudo anterior.^18^

A dimensão Capital Humano avalia oito fatores, com foco em educação/escolaridade, mortalidade infantil e mães jovens. Estudos nacionais prévios, identificaram que populações com menor escolaridade estavam mais predispostos a apresentar fatores de risco de DCVs e menor acesso a serviços de saúde.^39 , 40^ Foram descritos estudos que demonstram que maior mortalidade infantil pode estar associada a acesso mais difícil aos serviços de saúde.^18 , 41^ O exposto anteriormente pode justificar a melhor correlação desta dimensão com a mortalidade por DIC e DCBV. É provável que esta dimensão tenha alcançado grau de correlação superior ao próprio IVS, pois este sofreu influência das outras dimensões.

Por fim, a forte correlação da dimensão renda e trabalho, pode ser justificada pelo fato de o maior poder aquisitivo representar um acesso mais fácil a serviços de saúde. Estudos prévios identificaram que populações com menor poder aquisitivo têm mais fatores de risco cardiovasculares e maior mortalidade por DIC.^18 , 42^ Foi descrito também a relação inversa, uma vez que as DCVs podem deixar sequelas e incapacidades afetando a capacidade de trabalho do indivíduo e aumentando os gastos em saúde.^42^

O presente estudo traz uma metodologia inédita e contribui para o conhecimento atual das DCVs ao analisar as dimensões do IVS para a população total e para os estratos populacionais disponibilizados pelo IPEA, além de analisar o grau de correlação desses indicadores com a variação da taxa de mortalidade padronizada pela faixa etária de DIC e DCBV no Brasil e em suas UF.

Dentre as limitações do estudo estão o fato de se tratar de um estudo ecológico, que permite uma análise populacional preliminar que necessitará ser referendada por estudos prospectivos com coleta de dados sistematizada. As informações foram obtidas de banco de dados, podem estar vulneráveis a vieses por falhas na coleta de dados: subnotificação, causas mal definidas ou *garbage codes* . No entanto, se ressalta que tais limitações atuam de modo sistêmico, em todas as declarações e bancos de óbito, não sendo um impedimento para a análise global dos dados.

A hipótese central de que existiria correlação entre a diminuição da vulnerabilidade social e a redução das taxas de mortalidade por DIC e doenças cerebrovasculares foi corroborada nesse estudo. Analisar o IVS por estratos populacionais permite supor que o aglomerado populacional rural, as mulheres e os negros, historicamente associados com maior vulnerabilidade social, apresentariam menores reduções das taxas de mortalidade por DIC e DCBV. Novos estudos que contemplem períodos temporais maiores poderão auxiliar no entendimento de quais componentes do IVS causarão maior impacto na diminuição da morbimortalidade pelas DCVs, auxiliando no direcionamento de políticas públicas voltadas para a saúde.

## Conclusão

A análise das dimensões do IVS permitiu identificar a redução da vulnerabilidade do Brasil e da grande maioria das suas UF em todas as dimensões, apesar de persistirem as desigualdades com maior vulnerabilidade nas UF de Norte e Nordeste. Além disso, se identificou maior vulnerabilidade das populações negra e rural. Apesar da redução da vulnerabilidade nas UF de Norte e Nordeste, não houve redução mais expressiva de mortalidade por DIC e DCBV. Foi possível identificar correlação forte da variação do IVS, IVS-CH e IVS-RT com a variação da mortalidade por DIC e DCBV. Esses dados podem guiar investimentos públicos, com o intuito de reduzir a mortalidade por essas condições.

Disponibilidade de Dados

Os conteúdos subjacentes ao texto da pesquisa estão contidos no manuscrito.

## *Material suplementar

Para informação adicional, por favor, clique aqui


