# Supplementary material for: Índice de Vulnerabilidade Social e Mortalidade por Doenças Isquêmicas do Coração e Doenças Cerebrovasculares no Brasil de 2000 a 2021
Source: Arq Bras Cardiol. 2025 Aug 20;122(8):e20240428. [Article in Portuguese] doi: 10.36660/abc.20240428 (PMC12671727; doi:10.36660/abc.20240428)
Supplement: Supplementary file 2 [file 0066-782X-abc-122-08-e20240428-Suppl01-en.pdf]

SUPPLEMENTARY MATERIAL

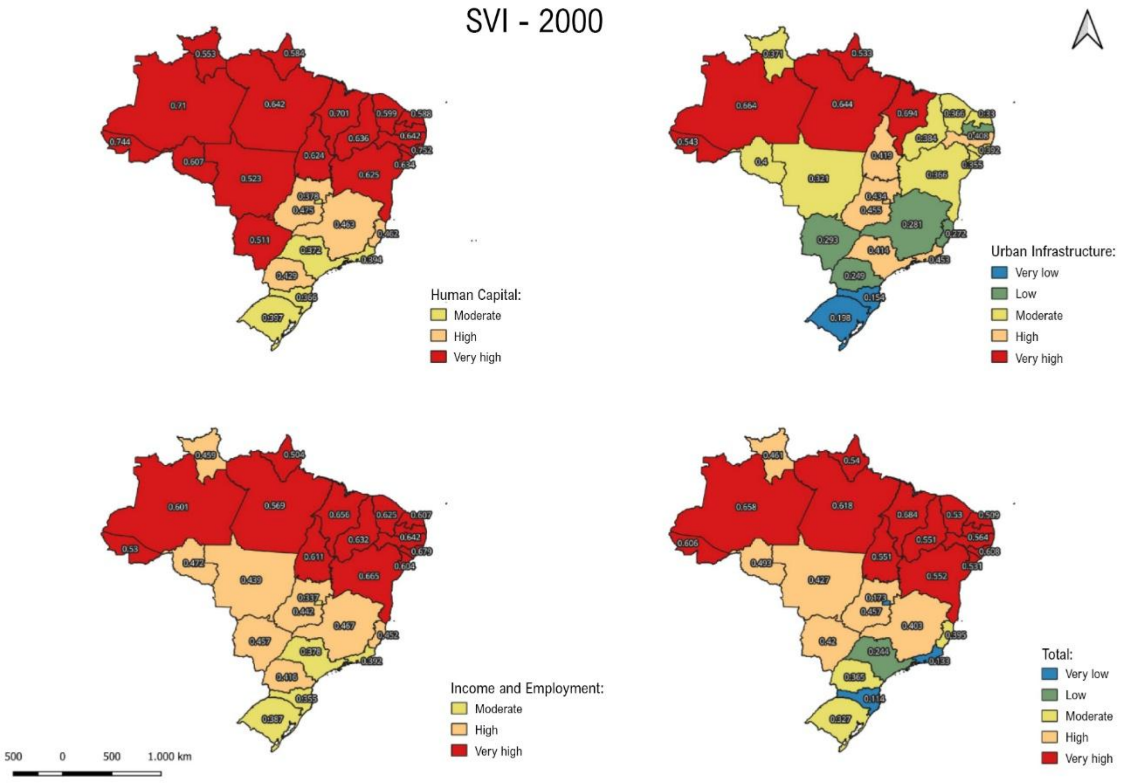

Supplementary Figure 1 - Maps with data from the SVI and its Human Capital, Urban Infrastructure, and Income and Employment dimensions across Brazilian FUs in 2000.

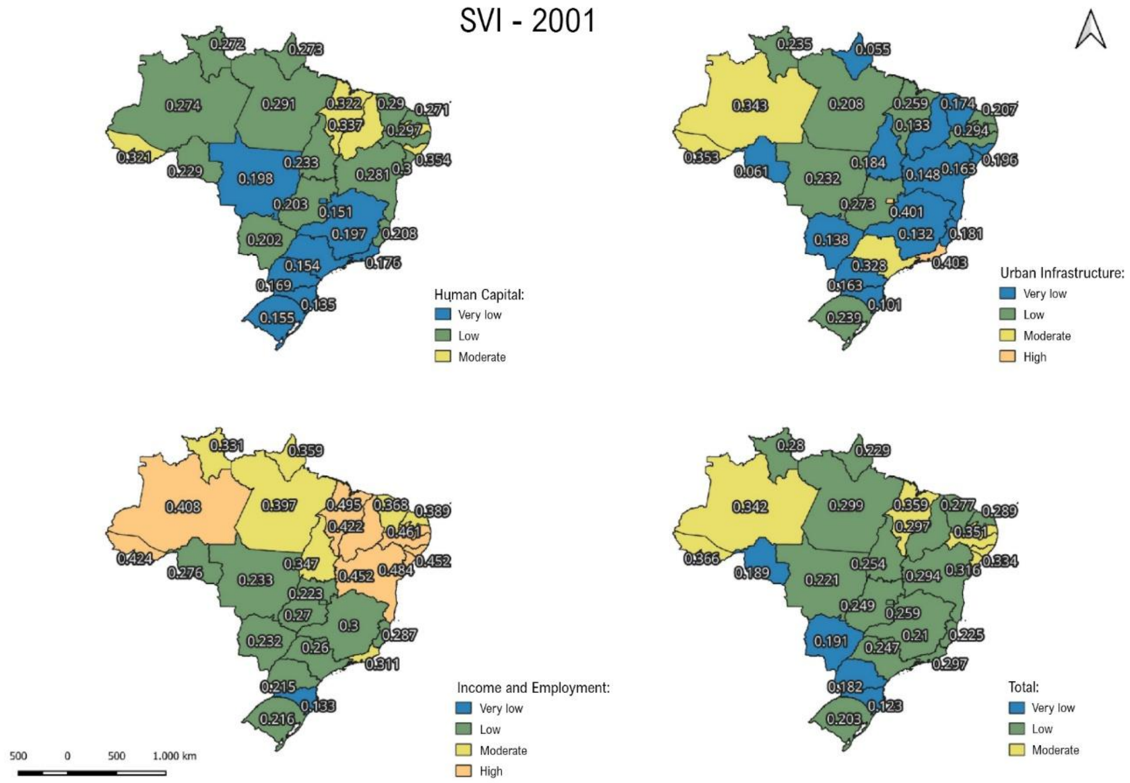

Supplementary Figure 2 - Maps with data from the SVI and its Human Capital, Urban Infrastructure, and Income and Employment dimensions across Brazilian FUs in 2021.

Supplementary Table 1 – Social Vulnerability Index in women, Black and White individuals, and urban populations

SVI	Women		Black		White		Urban	
	2000	2021	2000	2021	2000	2021	2000	2021
Brasil	0.419	0.263	0.502	0.283	0.335	0.207	0.390	0.238
Rondônia	0.466	0.19	0.504	0.194	0.404	0.18	0.408	0.174
Acre	0.574	0.341	0.591	0.371	0.516	0.322	0.510	0.334
Amazonas	0.603	0.338	0.642	0.354	0.530	0.273	0.565	0.33
Roraima	0.443	0.247	0.431	0.28	0.326	0.286	0.370	0.247
Pará	0.581	0.316	0.605	0.306	0.498	0.262	0.513	0.253
Amapá	0.491	0.242	0.523	0.237	0.427	0.185	0.469	0.225
Tocantins	0.517	0.249	0.547	0.266	0.430	0.2	0.488	0.244
Maranhão	0.632	0.346	0.659	0.368	0.562	0.317	0.591	0.317
Piauí	0.503	0.284	0.534	0.312	0.433	0.234	0.466	0.268
Ceará	0.481	0.294	0.522	0.285	0.425	0.258	0.456	0.264
Rio Grande do Norte	0.460	0.299	0.512	0.325	0.398	0.23	0.416	0.251
Paraíba	0.477	0.325	0.522	0.344	0.425	0.313	0.440	0.289
Pernambuco	0.500	0.347	0.556	0.367	0.451	0.316	0.478	0.331
Alagoas	0.547	0.33	0.598	0.344	0.493	0.301	0.517	0.301
Sergipe	0.486	0.312	0.517	0.321	0.417	0.292	0.440	0.281
Bahia	0.495	0.291	0.533	0.297	0.426	0.273	0.460	0.259
Minas Gerais	0.379	0.221	0.443	0.23	0.303	0.181	0.355	0.195
Espírito Santo	0.383	0.263	0.436	0.246	0.290	0.183	0.368	0.232
Rio de Janeiro	0.397	0.303	0.445	0.315	0.339	0.26	0.383	0.294
São Paulo	0.378	0.281	0.432	0.283	0.342	0.221	0.362	0.25
Paraná	0.352	0.194	0.433	0.212	0.315	0.17	0.328	0.172
Santa Catarina	0.281	0.123	0.405	0.165	0.252	0.111	0.254	0.112
Rio Grande do Sul	0.309	0.186	0.422	0.259	0.282	0.182	0.293	0.195
Mato Grosso do Sul	0.405	0.2	0.447	0.215	0.335	0.138	0.372	0.193
Mato Grosso	0.406	0.199	0.450	0.228	0.336	0.194	0.378	0.21
Goiás	0.439	0.28	0.484	0.264	0.373	0.205	0.425	0.25
Distrito Federal	0.369	0.27	0.403	0.277	0.314	0.229	0.355	0.258

Legend: Very high | High | Average | Low | Very low

Supplementary Table 2 – Urban Infrastructure dimension of the Social Vulnerability index in women, men, Black and White individuals, and urban populations.

SVI-UI	Women		Men		Black		White		Urban	
	2000	2021	2000	2021	2000	2021	2000	2021	2000	2021
Brasil	0.328	0.211	0.366	0.211	0.423	0.216	0.285	0.210	0.363	0.220
Rondônia	0.359	0.013	0.425	0.087	0.445	0.054	0.335	0.090	0.362	0.042
Acre	0.510	0.270	0.564	0.389	0.567	0.368	0.490	0.314	0.530	0.326
Amazonas	0.616	0.303	0.697	0.365	0.697	0.364	0.590	0.227	0.661	0.352
Roraima	0.359	0.108	0.381	0.300	0.373	0.215	0.265	0.364	0.292	0.161
Pará	0.596	0.211	0.669	0.211	0.663	0.208	0.538	0.215	0.564	0.168
Amapá	0.490	0.028	0.560	0.073	0.550	0.062	0.425	0.021	0.480	0.037
Tocantins	0.384	0.093	0.440	0.228	0.455	0.192	0.328	0.140	0.425	0.184
Maranhão	0.651	0.217	0.718	0.282	0.727	0.258	0.602	0.267	0.702	0.213
Piauí	0.353	0.086	0.403	0.154	0.412	0.140	0.308	0.092	0.399	0.127
Ceará	0.339	0.172	0.383	0.177	0.389	0.176	0.326	0.177	0.372	0.186
Rio Grande do Norte	0.308	0.221	0.344	0.204	0.371	0.234	0.270	0.159	0.279	0.161
Paraiba	0.275	0.181	0.311	0.226	0.322	0.178	0.264	0.298	0.261	0.181
Pernambuco	0.373	0.276	0.428	0.303	0.439	0.307	0.360	0.273	0.406	0.297
Alagoas	0.348	0.179	0.416	0.205	0.409	0.201	0.358	0.187	0.373	0.156
Sergipe	0.342	0.181	0.365	0.158	0.379	0.170	0.292	0.142	0.314	0.128
Bahia	0.338	0.135	0.385	0.156	0.384	0.155	0.305	0.118	0.366	0.125
Minas Gerais	0.256	0.131	0.297	0.132	0.336	0.132	0.211	0.134	0.288	0.125
Espírito Santo	0.270	0.245	0.274	0.147	0.324	0.184	0.181	0.176	0.326	0.220
Rio de Janeiro	0.451	0.398	0.456	0.404	0.477	0.405	0.435	0.385	0.451	0.403
São Paulo	0.413	0.391	0.415	0.288	0.422	0.358	0.411	0.300	0.411	0.342
Paraná	0.236	0.172	0.258	0.158	0.293	0.147	0.229	0.178	0.256	0.155
Santa Catarina	0.140	0.073	0.162	0.112	0.229	0.151	0.133	0.083	0.148	0.079
Rio Grande do Sul	0.180	0.154	0.209	0.292	0.257	0.282	0.181	0.205	0.205	0.229
Mato Grosso do Sul	0.267	0.113	0.311	0.155	0.322	0.143	0.242	0.071	0.273	0.148
Mato Grosso	0.281	0.139	0.346	0.276	0.371	0.235	0.258	0.192	0.312	0.220
Goiás	0.440	0.332	0.465	0.232	0.501	0.288	0.394	0.202	0.463	0.281
Distrito Federal	0.432	0.401	0.435	0.354	0.446	0.402	0.422	0.401	0.431	0.401

Legend: Very high | High | Average | Low | Very low

Supplementary Table 3 – Human Capital dimension of the Social Vulnerability Index in women, Black and White individuals, and rural and urban populations.

SVI-HC	Women		Black		White		Rural		Urban	
	2000	2021	2000	2021	2000	2021	2000	2021	2000	2021
Brasil	0.513	0.226	0.605	0.253	0.400	0.168	0.742	0.354	0.440	0.196
Rondônia	0.663	0.249	0.670	0.243	0.568	0.196	0.763	0.288	0.529	0.217
Acre	0.779	0.335	0.774	0.326	0.695	0.267	0.941	0.465	0.614	0.274
Amazonas	0.726	0.284	0.726	0.281	0.584	0.238	0.918	0.398	0.595	0.250
Roraima	0.569	0.284	0.546	0.281	0.413	0.232	0.827	0.304	0.474	0.269
Pará	0.675	0.306	0.682	0.305	0.558	0.220	0.848	0.452	0.539	0.234
Amapá	0.577	0.286	0.590	0.279	0.490	0.228	0.890	0.298	0.527	0.281
Tocantins	0.640	0.254	0.660	0.241	0.530	0.186	0.849	0.285	0.544	0.221
Maranhão	0.712	0.340	0.719	0.329	0.613	0.292	0.885	0.420	0.568	0.287
Piauí	0.655	0.355	0.674	0.363	0.554	0.228	0.819	0.407	0.539	0.308
Ceará	0.599	0.300	0.644	0.302	0.505	0.250	0.747	0.393	0.534	0.264
Rio Grande do Norte	0.574	0.268	0.635	0.308	0.490	0.211	0.762	0.388	0.508	0.243
Paraíba	0.623	0.327	0.680	0.353	0.534	0.255	0.766	0.462	0.562	0.275
Pernambuco	0.618	0.310	0.668	0.311	0.523	0.265	0.831	0.462	0.544	0.268
Alagoas	0.756	0.360	0.803	0.372	0.627	0.305	0.883	0.476	0.668	0.320
Sergipe	0.629	0.304	0.659	0.301	0.528	0.291	0.814	0.410	0.545	0.266
Bahia	0.622	0.296	0.640	0.280	0.513	0.279	0.794	0.373	0.519	0.248
Minas Gerais	0.480	0.210	0.546	0.218	0.383	0.165	0.654	0.288	0.417	0.182
Espírito Santo	0.498	0.211	0.557	0.226	0.380	0.163	0.643	0.289	0.430	0.196
Rio de Janeiro	0.404	0.187	0.479	0.201	0.310	0.142	0.626	0.348	0.379	0.171
São Paulo	0.396	0.156	0.495	0.181	0.337	0.135	0.569	0.220	0.369	0.151
Paraná	0.471	0.175	0.584	0.205	0.408	0.151	0.590	0.283	0.415	0.156
Santa Catarina	0.410	0.140	0.588	0.157	0.366	0.130	0.500	0.196	0.366	0.126
Rio Grande do Sul	0.425	0.163	0.582	0.201	0.380	0.145	0.495	0.214	0.391	0.148
Mato Grosso do Sul	0.552	0.211	0.600	0.227	0.437	0.170	0.784	0.270	0.476	0.194
Mato Grosso	0.562	0.194	0.592	0.203	0.457	0.187	0.764	0.246	0.479	0.191
Goiás	0.503	0.208	0.553	0.217	0.415	0.173	0.645	0.267	0.462	0.198
Distrito Federal	0.377	0.154	0.437	0.174	0.291	0.116	0.537	0.218	0.358	0.149

Legend: Very high | High | Average | Low | Very low

Supplementary Table 4 – Income and Employment dimension of the Social Vulnerability Index in women, men, Black and White individuals, and rural and urban populations.

SVI-IE	Women		Men		Black		White		Rural		Urban	
	2000	2021	2000	2021	2000	2021	2000	2021	2000	2021	2000	2021
Brasil	0.416	0.353	0.383	0.295	0.478	0.381	0.321	0.243	0.479	0.437	0.367	0.297
Rondônia	0.375	0.308	0.373	0.257	0.397	0.284	0.308	0.254	0.398	0.291	0.332	0.262
Acre	0.432	0.418	0.415	0.411	0.430	0.419	0.361	0.385	0.448	0.460	0.385	0.401
Amazonas	0.466	0.428	0.472	0.394	0.503	0.418	0.415	0.354	0.482	0.491	0.440	0.388
Roraima	0.401	0.349	0.353	0.322	0.374	0.344	0.299	0.263	0.434	0.415	0.343	0.312
Pará	0.471	0.432	0.449	0.381	0.468	0.406	0.399	0.352	0.461	0.474	0.435	0.357
Amapá	0.405	0.411	0.389	0.310	0.429	0.371	0.367	0.305	0.458	0.309	0.402	0.356
Tocantins	0.525	0.400	0.499	0.299	0.527	0.364	0.432	0.272	0.501	0.415	0.495	0.327
Maranhão	0.534	0.482	0.521	0.483	0.531	0.517	0.471	0.392	0.524	0.578	0.503	0.451
Piauí	0.501	0.411	0.508	0.435	0.515	0.432	0.438	0.380	0.540	0.521	0.459	0.368
Ceará	0.506	0.410	0.514	0.337	0.535	0.376	0.446	0.347	0.577	0.455	0.463	0.341
Rio Grande do Norte	0.496	0.408	0.490	0.380	0.530	0.432	0.435	0.320	0.558	0.542	0.461	0.349
Paraíba	0.532	0.468	0.541	0.459	0.565	0.502	0.476	0.387	0.579	0.619	0.498	0.411
Pernambuco	0.509	0.454	0.522	0.452	0.561	0.484	0.470	0.410	0.571	0.587	0.485	0.428
Alagoas	0.539	0.450	0.553	0.421	0.582	0.460	0.495	0.410	0.561	0.537	0.510	0.426
Sergipe	0.488	0.451	0.483	0.484	0.514	0.491	0.430	0.445	0.529	0.567	0.462	0.450
Bahia	0.527	0.441	0.536	0.431	0.574	0.456	0.460	0.422	0.578	0.572	0.495	0.405
Minas Gerais	0.401	0.321	0.369	0.285	0.446	0.340	0.315	0.244	0.437	0.407	0.359	0.279
Espírito Santo	0.380	0.335	0.370	0.247	0.425	0.329	0.309	0.211	0.438	0.306	0.347	0.281
Rio de Janeiro	0.336	0.323	0.299	0.272	0.381	0.339	0.272	0.254	0.397	0.395	0.320	0.309
São Paulo	0.326	0.296	0.283	0.228	0.380	0.311	0.277	0.227	0.352	0.347	0.305	0.256
Paraná	0.350	0.234	0.327	0.200	0.423	0.285	0.306	0.181	0.394	0.274	0.314	0.205
Santa Catarina	0.291	0.155	0.266	0.114	0.397	0.188	0.258	0.121	0.360	0.151	0.248	0.130
Rio Grande do Sul	0.323	0.241	0.295	0.196	0.428	0.293	0.285	0.197	0.396	0.264	0.284	0.208
Mato Grosso do Sul	0.397	0.276	0.361	0.196	0.417	0.274	0.325	0.174	0.373	0.197	0.366	0.235
Mato Grosso	0.377	0.264	0.344	0.214	0.387	0.245	0.292	0.204	0.354	0.264	0.284	0.208
Goiás	0.375	0.301	0.350	0.250	0.399	0.286	0.309	0.239	0.363	0.259	0.351	0.270
Distrito Federal	0.299	0.254	0.248	0.196	0.326	0.257	0.229	0.170	0.319	0.199	0.276	0.225

Legend: Very high | High | Average | Low | Very low
