# Supplementary material for: Índice de Vulnerabilidade Social e Mortalidade por Doenças Isquêmicas do Coração e Doenças Cerebrovasculares no Brasil de 2000 a 2021
Source: Arq Bras Cardiol. 2025 Aug 20;122(8):e20240428. [Article in Portuguese] doi: 10.36660/abc.20240428 (PMC12671727; doi:10.36660/abc.20240428)
Supplement: Translation

# Translation: Social Vulnerability Index and Mortality from Ischemic Heart Diseases and Cerebrovascular Diseases in Brazil from 2000 to 2021

**DOI:** 10.36660/abc.20240428i

**Authors:** José Lucas Bichara, Luiz Antônio Bastos, Eric Delgado dos Santos Mafra Lino, Paolo Blanco Villela, Gláucia Maria Moraes de Oliveira

**Affiliations:** 1 Rio de Janeiro RJ Brazil Universidade Federal do Rio de Janeiro, Rio de Janeiro, RJ – Brazil; 2 Rio de Janeiro RJ Brazil Instituto Militar de Engenharia, Rio de Janeiro, RJ – Brazil

**Keywords:** Myocardial Ischemia, Cerebrovascular Disorders, Cardiovascular Diseases, Social Vulnerability, Epidemiology

## Abstract

**Background:**

Previous studies have observed a correlation between mortality rates from ischemic heart diseases (IHDs), cerebrovascular diseases (CBVDs), and the Social Vulnerability Index (SVI). However, doubts persist about the association between the overall SVI and its dimensions and mortality stratified by sex, ethnicity, and population clusters.

**Objective:**

To analyze the evolution of the overall SVI and its dimensions and correlate it with mortality rates due to IHD and CBVD in Brazil and its Federative Units (FUs) from 2000 to 2021.

**Methods:**

Ecological study of time series of standardized mortality rates (using the direct method with the Brazilian population in 2000) due to IHD and CBVD categorized by age, sex, and FUs between 2000 and 2021, correlated with the SVI and its dimensions. Data on the underlying causes of death were obtained from the Mortality Information System, while SVI data were sourced from the Social Vulnerability Atlas. Spearman correlation (considered significant if p<0.05) was employed to calculate each analyzed stratum.

**Results:**

The SVI and its Human Capital (SVI-HC) dimension and Income and Employment (SVI-IE) dimension in 2010 showed a strong correlation with variations in mortality rates due to CBVD and IHD (SVI x CBVD: Rho(p)=0.85; SVI x IHD: Rho(p)=0.75; SVI-HC x CBVD: Rho(p)=0.84; SVI-HC x IHD: Rho(p)=0.84; SVI-IE x CBVD: Rho(p)=0.81; SVI-IE x IHD: Rho(p)=0.71). The Urban Infrastructure dimension (SVI-UI) showed a weak correlation with CBVD and IHD, respectively (SVI-UI x CBVD: Rho(p)=0.33; SVI-UI x IHD: Rho(p)=0.25).

**Conclusion:**

Both SVI-HC and SVI-IE demonstrated strong correlations with variations in mortality rates from IHD and CBVD.

## Introduction

In 2021, more than 9 million deaths were estimated to have occurred due to ischemic heart diseases (IHDs), and nearly 4 million deaths were attributed to ischemic stroke worldwide.^1^

Approximately 75% of these deaths were recorded in developing countries.^2 - 3^In Brazil, cardiovascular diseases are also the leading cause of death, primarily represented by IHDs and cerebrovascular diseases (CBVDs).^2 - 6^At the end of the 20th century, there was a faster reduction in mortality from these conditions in regions with improved socioeconomic indicators, such as Western Europe and North America.^7^In Brazil, the Federative Units (FUs) in the South, Southeast, and Central-West regions, which have the best socioeconomic indicators, showed greater reductions in mortality rates from IHD and CBVD.^6 , 8 , 9^

In recent years, there has been an increase in the number of studies associating socioeconomic indicators with mortality rates due to cardiovascular diseases; however, few studies have used vulnerability indicators in this analysis.^6 , 8 , 10 - 13^Despite this, these studies have in common the identification of higher mortality rates in more vulnerable populations.^6 , 8 , 10 - 13^

The term social vulnerability has several definitions; one of the most accepted is "the absence or insufficiency of assets that can be provided by the state."^14^On a global level, there is no standardization of the Social Vulnerability Index (SVI). In Brazil, the index is calculated by the Institute for Applied Economic Research ( *Instituto de Pesquisa Econômica Aplicada* , IPEA), which quantifies 16 indicators grouped into three dimensions: Urban Infrastructure, Human Capital, and Income and Employment (including the form of integration, whether formal or informal). Finally, the arithmetic mean of these three dimensions is calculated to yield the overall SVI value.^14^The Urban Infrastructure dimension reflects access to basic sanitation and urban transportation. The Human Capital dimension assesses a population's health conditions and access to education. Lastly, the Income and Employment dimension (including the form of insertion) identifies insufficient income and income flow.^14^The IPEA also provides calculations of the overall SVI and its dimensions for specific population segments, including women, men, Black and White individuals, and rural and urban populations. These data may help identify the most fragile characteristics in the most vulnerable populations.

The aim of this article was to analyze the evolution of the overall SVI and its dimensions, as well as its association with mortality rates due to IHD and CBVD in Brazil and its FUs from 2000 to 2021.

## Methods

Ecological and descriptive study of variations in mortality rates due to IHDs and CBVDs across both sexes and age groups between 2000 and 2021. The overall SVI and its dimensions in Brazil and its FUs between 2000 and 2021 were also analyzed.

Data on the underlying causes of death were obtained from the Mortality Information System ( *Sistema de Informações sobre Mortalidade* ; SIM) website maintained by the Information Technology Department of the Unified Health System ( *Departamento de Informática do Sistema Único de Saúde* ; DATASUS) within the Ministry of Health.^15^Mortality information for Brazil and its FUs was selected. Age group, sex, and deaths per residence were used as variables. For the research, the population was stratified into age groups as follows: 0–19 years, 20–29 years, and subsequently into 10-year bands up to the 80+ age group.

To select deaths with underlying causes of IHDs, International Classification of Diseases (ICD-10) codes I20–I25 were used. For CBVDs, the I60–I69.^16^codes were used. Subsequently, files in a CSV format were downloaded and converted into XLS using Microsoft Excel for data analysis and construction of graphs and tables.

Information on the population residing in Brazil and its FUs was obtained from the DATASUS website,^15^which uses official census data from the Brazilian Institute of Geography and Statistics ( *Instituto Brasileiro de Geografia e Estatística* ; IBGE) for 1980, 1991, 2000, and 2010, along with intercensal projections up to 2012, and population projections from 2013 onwards.

Information on SVI was obtained from the Atlas of Social Vulnerability provided by IPEA,^14^where the values of the SVI and its dimensions for both the overall population and the available population strata were collected. The SVI was considered very low when below 0.2, low when between 0.2 and 0.3, medium when between 0.3 and 0.4, high when between 0.4 and 0.5, and very high when greater than 0.5.^14^These values served as references for coloring the maps and tables in the supplementary material.

The crude and standardized mortality rates^17^were calculated by age group and sex using the direct method, with the Brazilian population of the year 2000 serving as the standard population for IHD and CBVD. The temporal trend of mortality rates standardized by age group and sex from 2000 to 2021 was evaluated, as well as the association with the SVI and its dimensions in 2010. The year 2021 was chosen as the cutoff because it is the most recent year for which data are available for all variables analyzed.

Tables were constructed using data from the overall SVI and its dimensions for the years 2000 and 2021. To construct the graphs, the percentage variations in standardized mortality rates for IHD and CBVD and the 2010 SVI were used. A time lag of approximately 10 years was adopted between the indicators and mortality rates, as per previous studies on this topic.^8 , 18 , 19^To analyze the degree of correlation, Spearman's correlation was calculated for each stratum analyzed, with significance set at p < 0.05. Correlations were considered weak when ≤ 0.3, moderate when > 0.3 and < 0.7, and strong when ≥ 0.7.^20^

## Results

From 2000 to 2021, there were 2,127,662 deaths from CBVDs and 2,193,405 deaths from IHDs in Brazil, with 50.58% and 58.29%, respectively, attributed to the male sex. During the same period, the SVI in Brazil ranged from 0.446 in 2000 to 0.249 in 2021.

The analysis of the SVI and its dimensions within the overall population and across the available population strata revealed improvements in this indicator in nearly all FUs. However, throughout the entire period, the best indicators were concentrated in the FUs of the South, Southeast, and Central-West regions ( Supplementary Figures 1 and 2 and Tables 1, 2, 3, and 4 ). It was also clear that, in 2000, almost all FUs with very high vulnerability were situated in the North and Northeast regions of the country ( Supplementary Figure 1 and Tables 1, 2, 3, and 4 ). Another key fact to highlight is that the Black and rural populations exhibited the highest vulnerability compared with the other segments analyzed ( Supplementary Tables 1, 2, 3, and 4 ).

The Urban Infrastructure (SVI-UI) dimension exhibited lower vulnerability than the overall SVI and demonstrated the highest number of FUs classified as having low and very low vulnerability in 2021 ( Supplementary Table 2 ). A greater number of FUs were classified as having very high vulnerability in the Human Capital (SVI-HC) dimension, primarily in the year 2000, encompassing all FUs in the North and Northeast. Furthermore, Black and rural populations presented greater vulnerability than other population segments ( Supplementary Table 3 ). Finally, the Income and Employment (SVI-IE) dimension highlighted greater vulnerability in the female population compared with the male population ( Supplementary Table 4 ).

As shown in Figure 1 , the populations of the FUs in the North and Northeast regions exhibited greater vulnerability and either a worsening or less substantial reduction in mortality rates due to IHD and CBVD. Notably, the FUs in the South region demonstrated lower vulnerability with a more significant reduction in mortality from IHD and CBVD. Spearman's correlation was calculated for all scenarios, revealing a moderate to strong correlation. The correlation coefficients found were: SVI x CBVD, Rho(p)=0.85; SVI x IHD, Rho(p)=0.75.

**Figure 1:** Comparison of the SVI in the year 2010 and the percentage variation in the mortality rate due to cerebrovascular diseases and ischemic heart diseases in Brazil and its Federative Units, for both sexes, from 2000 to 2021. (1A) - 2010 SVI and percentage variation in mortality from CBVD, for both sexes, in Brazil, from 2000 to 2021. (1B) - 2010 SVI and percentage variation in mortality from IHD, for both sexes, in Brazil, from 2000 to 2021.

Figure 2 illustrates that the FUs in the North and Northeast regions displayed higher vulnerability in the SVI-UI dimension than the FUs in the South region. Furthermore, the FUs in the North and Northeast regions exhibited a worsening or less expressive reduction in mortality due to IHD and CBVD. However, when the Central-West and Southeast regions were evaluated, it was observed that the Federal District, Goiás, Rio de Janeiro, and São Paulo exhibited greater vulnerability than most of the FUs in the North and Northeast regions. Spearman's correlation revealed a weak correlation. The correlation coefficients found were: SVI-UI x CBVD, Rho(p)=0.33; SVI-UI x IHD, Rho(p)=0.25.

**Figure 2:** Comparison of the Urban Infrastructure dimension of the 2010 Social Vulnerability Index and the percentage variation in the mortality rate from cerebrovascular diseases and ischemic heart diseases in Brazil and its Federative Units, for both sexes, from 2000 to 2021. (2A) - 2010 SVI-UI and percentage variation in mortality due to CBVD, in both sexes, in Brazil, from 2000 to 2021. (2B) - 2010 SVI-UI and percentage variation in mortality rates due to IHD, for both sexes, in Brazil, from 2000 to 2021.

In Figure 3 , the pattern was similar to that observed in Figure 1 , where the FUs in the North and Northeast regions exhibited greater vulnerability in terms of human capital, alongside a worsening or less pronounced reduction in mortality due to IHD and CBVD compared with the other regions. Notably, Spearman's correlation identified a strong correlation between SVI-HC and the others. The correlation coefficients were: SVI-HC x CBVD, Rho(p)=0.84; SVI-HC x IHD, Rho(p)=0.84.

**Figure 3:** Comparison of the Human Capital dimension of the 2010 Social Vulnerability Index and the percentage variation in mortality rate from cerebrovascular diseases and ischemic heart diseases in Brazil and its Federative Units, for both sexes, from 2000 to 2021. (3A) – 2010 SVI-HC and percentage variation in mortality due to CBVD, for both sexes, in Brazil, from 2000 to 2021. (3B) - 2010 SVI-HC and percentage variation in mortality due to IHD, for both sexes, in Brazil, from 2000 to 2021.

In Figure 4 , the pattern observed in Figures 1 and 3 reappears. The FUs in the North and Northeast regions exhibited higher vulnerability in the SVI-IE dimension and demonstrated either an increase in mortality due to IHD and CBVD, or, in the case of a decrease, this was less pronounced compared with other FUs in the other regions. Spearman's correlation identified a strong correlation, although it was less pronounced than the values obtained for SVI-HC and the SVI. The correlation coefficients were: SVI-IE x CBVD, Rho(p)=0.81; SVI-IE x IHD, Rho(p)=0.71.

**Figure 4:** Comparison of the Income and Employment dimension of the 2010 Social Vulnerability Index and the percentage variation in mortality rate from cerebrovascular diseases and ischemic heart diseases in Brazil and its Federative Units, for both sexes, from 2000 to 2021. (4A) - 2010 SVI-IE and percentage variation in mortality due to CBVD, for both sexes, in Brazil, from 2000 to 2021. (4B) - 2010 SVI-IE and percentage variation in mortality rate due to IHD, for both sexes, in Brazil, from 2000 to 2021.

## Discussion

In assessing the SVI dimensions, it was observed that despite improvements in indicators during the period, the vast majority of the FUs in the North and Northeast remained the most vulnerable in the country across all dimensions. Furthermore, there was a clear greater vulnerability among Black and rural populations, as well as increased vulnerability in the Human Capital dimension of the SVI. These findings were also described in other studies observing greater vulnerability among Black populations^21 - 23^and rural populations^24 - 26^in several areas, including access to healthcare.

It was observed that there was either an increase in mortality from IHD and CBVD, or any decrease observed was less pronounced in the FUs of the North and Northeast regions compared with the other FUs, despite the reduction in vulnerability observed during this period. The analysis of the correlations between the variation in mortality due to IHD and CBVD with the dimensions of the SVI alone revealed that the SVI-HC (> 0.83 – strong) achieved a stronger correlation than the SVI (> 0.75 – strong). Additionally, SVI-IE also showed a strong correlation, while SVI-UI exhibited a weak correlation (< 0.35). Previous studies have already identified an association between socioeconomic indicators of development and vulnerability and mortality from these conditions.^6 , 8 , 9 , 18 , 27 - 31^However, no analysis has yet been conducted to identify which factors have the greatest influence.

Regarding vulnerability, studies from the United States suggest that vulnerability metrics may correlate well with public health outcomes.^31^International studies have identified that populations with greater vulnerability are more likely to have cardiovascular risk factors such as hypertension, dyslipidemia, diabetes and smoking,^10 , 32^greater difficulty in accessing healthcare services,^33^less access to cardiac rehabilitation services,^33 , 34^more hospital readmissions within 30 days due to heart failure,^35^and increased mortality, including early mortality due to cardiovascular diseases.^12 , 13^

Previous studies analyzing the SVI in Brazil have identified improvements at a national level but greater vulnerability in the North and Northeast regions,^6 , 8^which aligns with our findings. Concerning mortality due to CBVD, a decline in mortality was also observed in the FUs of the South, Southeast, and Central-West regions,^6^which was similar for IHD.^8^This study suggests the socioeconomic factors that may have the greatest impact on mortality from both IHD and CBVD.

The Urban Infrastructure dimension, which evaluates garbage collection, inadequate water and sewage supply, and commuting times, demonstrated a weaker correlation than the other dimensions. These findings are primarily due to the fact that the FUs of Federal District (DF), Goiás (GO), Rio de Janeiro (RJ), and São Paulo (SP) exhibited greater vulnerability, contrasting with the patterns observed in the other dimensions. It can be assumed that in RJ and SP, this phenomenon is attributed to the large migratory movements that began in the 1950s, which resulted in rapid and disorganized growth of the urban population, leading to the formation of slums ( *favelas* ).^36 , 37^The FUs of GO and DF also experienced a similar process, though on a smaller scale, starting in the 1960s, motivated by the construction of the Federal Capital and later by the expansion of the agricultural frontier.^38^

Other possible explanations for the correlations not being strong for the SVI-UI dimension include the need for a longer time lag between the improvements in this dimension of social vulnerability and a reduction in mortality, or that this dimension may be represented by other SVI dimensions, such as the Human Capital, which measures infant mortality and years of schooling, or the Income and Employment, which assesses per capita household income, which has already shown isolated correlation in a previous study.^18^

The Human Capital dimension assesses eight factors related to education/schooling, infant mortality, and young mothers. Previous national studies have identified that populations with lower levels of education are more likely to exhibit risk factors for cardiovascular disease and have less access to healthcare services.^39 , 40^Studies have also shown that higher infant mortality may be associated with more difficult access to healthcare services.^18 , 41^This may justify the stronger correlation of this dimension with mortality from IHD and CBVD. It is likely that this dimension achieved a higher degree of correlation than the SVI itself, as the other dimensions influenced the latter.

Finally, the strong correlation observed in the Income and Employment dimension may be explained by the fact that greater purchasing power leads to access to healthcare services. Previous studies have identified that populations with lower purchasing power have more cardiovascular risk factors and higher mortality due to IHD.^18 , 42^An inverse relationship has also been described, as cardiovascular diseases can leave sequelae and disabilities, affecting the individual's ability to work and increasing healthcare costs.^42^

The present study employs a novel methodology and enhances current knowledge of cardiovascular diseases by analyzing the dimensions of the SVI for the overall population and for the population strata provided by the IPEA. Additionally, it examines the degree of correlation between these indicators and the variation in the age-standardized mortality rates for IHD and CBVD in Brazil and its FUs.

The limitations of the study include the fact that it is an ecological study, which permits a preliminary population analysis that requires validation by future prospective studies with systematic data collection. The data were obtained from databases and may be susceptible to bias due to issues in data collection, including underreporting, poorly defined causes, or garbage codes. However, it is important to note that these limitations are systemic across all death certificates and databases and do not hinder the global analysis of the data.

The central hypothesis of a correlation between a reduction in social vulnerability and a decrease in mortality rates from IHDs and CBVDs was corroborated in this study. Analyzing the SVI by population strata allowed us to infer that the clusters of rural populations, women, and Black individuals, historically associated with greater social vulnerability, would experience smaller reductions in mortality rates due to IHD and CBVD. Future studies covering longer time periods may improve the understanding of which components of the SVI will have the greatest impact on reducing morbidity and mortality from cardiovascular diseases, helping to guide public policies focused on health.

## Conclusion

The analysis of the SVI dimensions enabled the identification of a reduction in vulnerability in Brazil and in most of its FUs across all dimensions, despite the persistence of inequalities with greater vulnerability in the FUs in the North and Northeast. Additionally, increased vulnerability was identified among Black and rural populations. Despite the reduction in vulnerability in the North and Northeast FUs, a pronounced decrease in mortality from IHDs and CBVDs was not observed. A strong correlation was identified between the variation in SVI, SVI-HC, and SVI-IE and the variation in mortality from IHD and CBVD. These data may inform public investments aimed at reducing mortality from these conditions.

Data Availability

The underlying content of the research text is contained within the manuscript.

## *Supplemental Materials

For additional information, please click here
